# Associations between degrees of task delegation and job satisfaction of general practitioners and their staff: a cross-sectional study

**DOI:** 10.1186/s12913-017-1984-y

**Published:** 2017-01-17

**Authors:** Helle Riisgaard, Jens Søndergaard, Maria Munch, Jette V. Le, Loni Ledderer, Line B. Pedersen, Jørgen Nexøe

**Affiliations:** 1 Research Unit of General Practice, Department of Public Health, University of Southern Denmark, J.B. Winsløws Vej 9A, Odense C, 5000 Denmark; 2 Section of Health Promotion and Health Services, Department of Public Health, Aarhus University, Bartholins Allé 2, Aarhus C, 8000 Denmark; 3 COHERE, Department of Business and Economics, University of Southern Denmark, Campusvej 55, Odense M, 5230 Denmark

**Keywords:** Task delegation, Job satisfaction, Staff’s role, General practice, Cross-sectional study

## Abstract

**Background:**

In recent years, the healthcare system in the western world has undergone a structural development caused by changes in demography and pattern of disease. In order to maintain the healthcare system cost-effective, new tasks are placed in general practice urging the general practitioners to rethink the working structure without compromising the quality of care. However, there is a substantial variation in the degree to which general practitioners delegate tasks to their staff, and it is not known how these various degrees of task delegation influence the job satisfaction of general practitioners and their staff.

**Methods:**

We performed a cross-sectional study based on two electronic questionnaires, one for general practitioners and one for their staff. Both questionnaires were divided into two parts, a part exploring the degree of task delegation regarding management of patients with chronic obstructive pulmonary disease in general practice and a part concerning the general job satisfaction and motivation to work.

**Results:**

We found a significant association between perceived “maximal degree” of task delegation in management of patients with chronic obstructive pulmonary disease and the staff’s overall job satisfaction. The odds ratio of the staff’s satisfaction with the working environment displayed a tendency that there is also an association with “maximal degree” of task delegation. In the analysis of the general practitioners, the odds ratios of the results indicate that there is a tendency that “maximal degree” of task delegation is associated with overall job satisfaction, satisfaction with the challenges in work, and satisfaction with the working environment.

**Conclusions:**

We conclude that a high degree of task delegation is significantly associated with overall job satisfaction of the staff, and that there is a tendency that a high degree of task delegation is associated with the general practitioners’ and the staff’s satisfaction with the working environment as well as with general practitioners’ overall job satisfaction and satisfaction with challenges in work. To qualify future delegation processes within general practice, further research could explore the reasons for our findings.

**Electronic supplementary material:**

The online version of this article (doi:10.1186/s12913-017-1984-y) contains supplementary material, which is available to authorized users.

## Background

In recent years, the healthcare system in the western world has undergone a structural development caused by changes in demography and pattern of disease. The population is ageing, and chronic disease is increasingly prevalent imposing a considerable burden upon society [1]. In order to maintain the healthcare system cost-effective, new tasks are placed in general practice, such as various clinical functions formerly undertaken in outpatient clinics [2, 3]. Consequently, general practitioners (GPs) are urged to rethink the working structure without compromising the quality of care.

It is a common assumption that delegating tasks from GPs to their staff is an appropriate way to deal with the continuously increasing workload in general practice [2, 4]. Task delegation is defined as an intentional transfer of clinical tasks from the GP to another healthcare professional, or another employee with clinical training, within the staff.

Research shows that the healthcare staff is generally capable of managing certain clinical tasks that were previously the domain of GPs, and that they are able to do this with a medical quality of care equivalent to the GPs’ [4–6]. However, this has primarily been investigated with regard to nurses. Additionally, patient satisfaction is found to be as high or higher with nurse-led care [4, 7]. Further, nurses in primary care have been shown to have potential to play an essential part in the management of chronic disease and complex conditions [1].

Quality is traditionally perceived as the medical quality of care provided by healthcare professionals or as the quality of care perceived by the patients [8]. However, job satisfaction may be another important quality parameter since it has shown to be associated with the medical quality of care in certain areas of healthcare [9]. Moreover, associations have been found between non-physicians’ job satisfaction and patient satisfaction with the care provided to them [10, 11]. Job satisfaction has been defined as the affective orientation one has towards his or her job, either as a global feeling about the job or as a related constellation of attitudes to various aspects or facets of the job, for example co-workers, personal growth and the nature of the work itself [12].

Few qualitative studies and one study using a quantitative study design have investigated whether practice nurses working more independently may free up GP time and improve GPs’ and their staff’s job satisfaction [13–17]. These studies indicate that task delegation and job satisfaction are important aspects of the organisation of general practice. However, there is a substantial variation in the degree to which general practitioners delegate tasks to their staff, and it is not known how these various degrees of task delegation influence the job satisfaction of GPs and their staff. Therefore, with our study, we aimed to contribute to the knowledge about GPs’ and their staff’s job satisfaction, especially with a view to examining if there is an association between degrees of task delegation and job satisfaction. According to a Medline search, our study is the first to quantitatively investigate the relationship between degrees of task delegation and GPs’ and their staff’s job satisfaction.

Hackman and Oldham developed a theoretical model explaining how certain working conditions motivate individuals in an organisation [18]. The model consists of five core job dimensions leading to three psychological states. The five core job dimensions are skill variety, task identity, task significance, autonomy and feedback. Skill variety means the degree to which a job requires various skills in carrying out the work. Task identity is the degree to which the job requires to have an overview of the complete unit of service. Task significance is the degree to which the job is meaningful. Autonomy is the freedom of scheduling and performing one’s own work. Feedback is the individual’s possibility of obtaining information about one’s performance. The three psychological states are meaningfulness of work, autonomy leading to experienced responsibility for outcomes of the work, and feedback which is the way an employee can obtain knowledge of how well he or she is performing in the job. Each psychological state contributes to high internal work motivation, high quality work performance, high satisfaction with the job, and low absenteeism and turnover. Jacobsen and Thorsvik [19] have elaborated on the model by emphasising that delegating tasks is a structural feature of an organisation which is characterised by the core job dimension, autonomy. According to the theoretical model, task delegation and job satisfaction appear to be associated.

Inspired by this model, we aimed to apply the conceptual framework of the model to the setting of Danish general practice exploring how certain features of the work might influence GPs’ and their staff’s psychological states and lead to job satisfaction. Thus, the objective of this study was to investigate associations between degrees of task delegation and job satisfaction of GPs and their staff in Danish general practice using the management of patients with chronic obstructive pulmonary disease (COPD) as our case.

## Methods

### Setting

General practice in Denmark comprises approximately 3600 GPs distributed on 2200 clinics. The majority are working in partnership practices which are increasing in number, while the number of singlehanded practices decreases. The GPs are self-employed, but work on contract for the public funder. The majority of practices employ ancillary staff, most frequently nurses and medical secretaries [20], but the care for the patients remains the overall responsibility of the GPs.

### Survey design

The study was based on two electronic questionnaires, one for GPs and one for their staff. The questionnaires were similar except for few questions especially designed for GPs or for staff. Both questionnaires were divided into two parts, a part exploring the degree of task delegation regarding management of patients with COPD in general practice and a part concerning the general job satisfaction and motivation to work.

In the part concerning task delegation, various tasks regarding management of patients with COPD were listed under the different consultation types in which they were performed, and the GPs as well as their staff were asked to state who was typically undertaking each task in their clinic. See the questions and the response categories in Table 1 and the questions as they appeared in the questionnaire in the Additional file 1.

**Table 1:** Questions asked in both the GP and the staff questionnaire

Questions	Response categories
Task delegation	
Who is typically undertaking the following task regarding diagnosing of patients with COPD in your practice? Select one or more answers	GP, including GP trainee/Nurse/Medical laboratory technician/Secretary or other staff member Not performed/Performed elsewhere/Do not know
Who is typically undertaking the following task regarding annual check-ups of patients with COPD in your practice? Select one or more answers	GP, including GP trainee/Nurse/Medical laboratory technician/Secretary or other staff member Not performed/Performed elsewhere/Do not know
Who is typically undertaking the following task regarding semiannual and quarterly check-ups of patients with COPD in your practice? Select one or more answers	GP, including GP trainee/Nurse/Medical laboratory technician/Secretary or other staff member Not performed/Performed elsewhere/Do not know
Who is typically undertaking the following task regarding exacerbations in patients with COPD in your practice? Select one or more answers	GP, including GP trainee/Nurse/Medical laboratory technician/Secretary or other staff member Not performed/Performed elsewhere/Do not know
Job satisfaction	
How satisfied are you with the challenges in your work?	Very satisfied/satisfied/unsatisfied/very unsatisfied
How satisfied are you with the general working environment in your practice?	Very satisfied/satisfied/unsatisfied/very unsatisfied
How satisfied are you with your job as a whole, everything taken into consideration?	Very satisfied/satisfied/unsatisfied/very unsatisfied

In the part concerning job satisfaction and motivation to work, the GPs as well as their staff were asked questions regarding the challenges in their work, the general working environment in the practice, and their overall job satisfaction (see questions and response categories in Table 1).

We tested both questionnaires in four steps. First, a pre-pilot study was conducted involving persons who did not belong to the target population assessing comprehensibility of the questionnaire. The GP questionnaire was assessed by 14 persons, and 17 persons assessed the staff questionnaire. The questionnaires were revised according to the pre-pilot study. Second, a pilot study was performed including 9 GPs testing relevance, acceptability and feasibility as well as comprehensibility and completeness in the GP questionnaire. In the staff questionnaire, these features were tested by 13 nurses. Third, we performed a qualitative pilot test inspired by “The three step test interview” [21] involving five GPs and five nurses. Fourth, in order to further qualify the questionnaires, and if possible reach consensus on the content, we performed two focus group interviews. One group discussed the GP questionnaire, and the other group debated the questionnaire for the staff. Both groups consisted of four persons including healthcare professionals as well as laymen. Minor revisions were made before the questionnaires were distributed to their respective target groups. Key questions remained unchanged in the two questionnaires.

### Participants

The GP questionnaire was distributed by e-mail on 4th December 2013 to all Danish GPs who then had an e-mail address registered at the Organisation of General Practitioners in Denmark (*n* = 3440). A reminder was sent out on 7th January 2014.

Since the study was targeted the management of patients with COPD, a question was added in the GP questionnaire concerning how many employees in the clinic were managing patients with this disease. Based on the GPs’ answers to this question, the staff in each practice afterwards received a letter containing a number of personal codes for the online questionnaire according to the number stated by the GPs. In case of disagreement between GPs within the same practice, the highest number stated was sent out.

The staff questionnaire was distributed by mail on 27th March 2014 to 2169 members of the staff in the practices that had participated in the GP survey. A reminder was sent out on 22nd May 2014.

### Measures

The outcome variables were job satisfaction of GPs and their staff measured on an individual level using a 4-point Likert scale. The explanatory variable was task delegation encompassing three categories according to three degrees of task delegation in general practice.

The question about overall job satisfaction was picked out from The Copenhagen Psychosocial Questionnaire (COPSOQ) [22], and the rest of the job satisfaction questions were in accordance with themes identified in preceding interviews and inspired by similar questions asked in COPSOQ. In order to explore associations between degrees of task delegation and job satisfaction, we developed a task delegation variable categorised into various degrees of task delegation by means of interviews, since we did not find any existing categorisation suitable for our study.

The preceding interviews did not only serve the purpose of qualifying the content of the job satisfaction questions, but were also targeted at identifying the types of consultations regarding management of COPD patients which could be conducted in general practice and the tasks which could be delegated from the GP to the staff in relation to these consultations.

Four types of consultations were identified: diagnosing, annual follow-ups, semi-annual/quarterly follow-ups and exacerbations and, in relation to each of them, a number of clinical tasks. The majority of the tasks appeared in more than one type of consultations, and a few of them appeared in only one consultation type. See the identified tasks in the Additional file 2.

To qualify the categorisation of the task delegation variable, we conducted further interviews with three GPs, two nurses and one healthcare worker. For the three consultation types, diagnosing, annual follow-ups and exacerbations, the informants were asked to assess the tasks identified in the previous interviews and rate them in order to determine their complexity with regard to delegation to the staff, 1 being minor tasks, 2 more complex tasks and 3 major tasks. We did not include tasks performed in semi-annual/quarterly follow-ups in this assessment since not all practices are performing those types of consultations. After the rating of the tasks, the informants were asked to explain their assessment, and based on this we could derive themes determining the graduation of task delegation. The level of independence and responsibility regarding assessment and decision making in the management of patients were common themes in the interviews, thus defining the categories (see Table 2). We categorised task delegation into three degrees: “minimal degree”, “medium degree” and “maximal degree”.

**Table 2:** Definition of degrees of task delegation and the distribution of the GP and staff population on these degrees

Degree of task delegation	Definition of the degree of task delegation	Content of delegated tasks	GPs *N* (%)	Staff *N* (%)
Minimal degree:	No responsibility for assessment in treatment or for decision making regarding further treatment.	Staff manages laboratory tasks and clinical procedures such as drawing blood samples and measuring blood pressure.	832 (66.83)	355 (56.26)
Medium degree:	Delegated responsibility for assessment in treatment, but no responsibility for decision making regarding further treatment.	Staff performs more complex tasks such as assessment of functional level, e.g. using an MRC scale, or manages independent consultations, e.g. counselling with regard to smoking cessation or diet and exercise.	294 (23.61)	164 (25.99)
Maximal degree:	Delegated responsibility for assessment in treatment and/or decision making regarding further treatment.	Staff performs highly complex tasks such as assessment of needs for initiating or adjusting COPD medication or assessment of indication for use of antibiotics.	119 (9.56)	112 (17.75)
Total			1245 (100)	631 (100)

Since no staff in general practice is performing tasks belonging to only one of the three categories, we developed an algorithm for the distribution of respondents into each of the categories. Based on the assumption that there are very few practices in which the staff is responsible for performing highly complex tasks independently, answering that minimum one task rated as 3 was performed by the staff would automatically place the respondent’s perceived degree of task delegation in the “maximal degree” category. In order to determine the distribution of the rest of the respondents’ perception of the degree of task delegation in their clinics, we had to decide how many tasks rated as 2 would be a reasonable minimum for being distributed to the “medium degree” category. Eight tasks across the various consultation types were rated as 2, and the rest was rated as 1. We assumed that one and two tasks rated as 2 would be an unrealistic low number for being placed in the “medium degree” category, and therefore, the threshold should be minimum three tasks. The remaining respondents having answered that two or less tasks rated as 2 were undertaken by the staff were placed in the “minimum degree” category. This distribution of task delegation was used as the explanatory variable in the analyses of associations between the degree of task delegation and job satisfaction. The distribution of task delegation as perceived by the respondents is shown in Table 2.

### Data analysis

The analyses explored associations between *perceived* degree of task delegation and job satisfaction on an individual level. This means that individuals within the same practice could be allocated into different categories depending on their perception of the degree of task delegation in the clinic.

There was a pronounced ceiling effect regarding the four included outcome variables (see Table 3). Therefore, we used a mixed-effect ordered logit model in the analyses with clustering on practice level in order to avoid leaving out important information.

**Table 3:** Distribution of job satisfaction of GPs and their staff

		Satisfaction			
Healthcare professional	Satisfaction variable	Very satisfied	Satisfied	Unsatisfied	Very Unsatisfied
GPs *N* = 1245	Overall satisfaction	423 (33.98%)	724 (58.15%)	88 (7.07%)	10 (0.80%)
	Challenges in work	595 (47.79%)	600 (48.19%)	46 (3.69%)	4 (0.32%)
	Working environment	538 (43.21%)	617 (49.56%)	78 (6.27%)	12 (0.96%)
Staff *N* = 631	Overall satisfaction	291 (46.12%)	322 (51.03%)	18 (2.85%)	0 (0%)
	Challenges in work	289 (45.80%)	318 (50.40%)	22 (3.49%)	2 (0.32%)
	Working environment	215 (34.07%)	350 (55.47%)	60 (9.51%)	6 (0.95%)

In both analyses, we adjusted for practice type, age and time pressure. Additionally, we adjusted for occupation in the staff analysis and gender in the GP analysis. Potential clustering within the practices was taken into account in both analyses.

We used “medium degree” of delegation as our reference group. This decision was based on the interviews with GPs and their staff developing the hypothesis that this way of working is the most commonly preferred in general practice. *P*-values ≤ 0.05 were considered statistically significant.

## Results

Of the 3440 invited GPs, 1580 responded to the questionnaire corresponding to 46.4%. Of the 1086 participating practices, 969 practices reported to have staff managing COPD patients. A total of 1245 GPs answered all questions of relevance for this study.

**Fig. 1:**
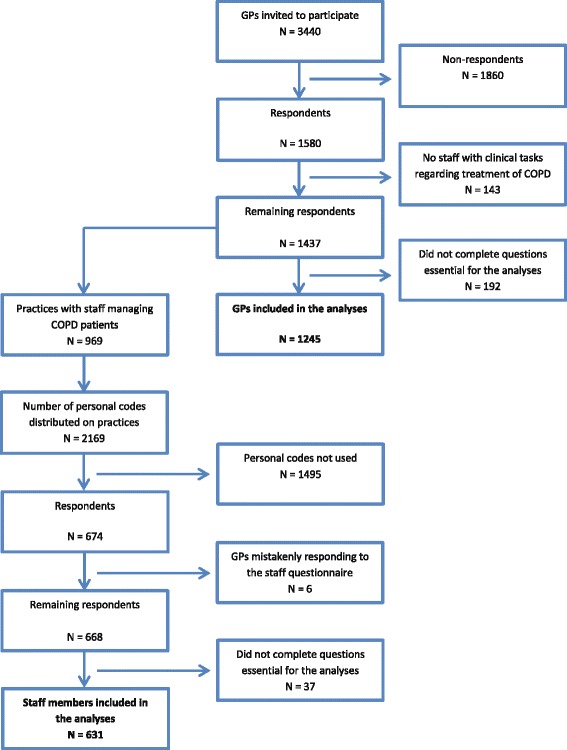
Inclusion of GPs and staff members in the study

Overall, 668 staff members distributed on 430 practices responded to the questionnaire corresponding to 39.6% of the participating practices. Of these, 631 respondents (from 409 practices) answered all questions in the questionnaire. Figure 1 shows a flow chart of the inclusion of GPs and staff members in the study.

### Task delegation and the staff’s job satisfaction

The staff’s overall job satisfaction showed a significant association with “maximal degree” of task delegation compared to “medium degree” (OR = 1.88, *p*-value = 0.048). The results regarding satisfaction with the general working environment showed a positive association, although only significant on a 10% significance level. This result displays a tendency that there is an association between “maximal degree” of task delegation and the staff’s satisfaction with the working environment (OR = 4.33, *p*-value = 0.094). We found no associations between degrees of task delegation and satisfaction with challenges in work, neither at a 5%, nor at a 10% level (see Table 4).

**Table 4:** Associations between degrees of delegation and job satisfaction of GPs and their staff

		Job satisfaction					
Healthcare professional	Degree of delegation	Overall job satisfaction		Challenges in work		Working environment	
		OR adj. (95% CI)	*p*	OR adj. (95% CI)	*p*	OR adj. (95% CI)	*p*
Staff *N* = 631	Minimal	1.23 (0.75;2.02)	0.401	0.98 (0.62;1.55)	0.935	2.21 (0.62;7.86)	0.222
	Medium	1	-	1		1	
	Maximal	1.88 (1.00;3.51)^**^	0.048	1.26 (0.71;2.21)	0.431	4.33 (0.78;24.08)^*^	0.094
GPs *N* = 1245	Minimal	1.08 (0.80;1.45)	0.618	1.00 (0.76;1.31)	0.976	1.03 (0.75;1.42)	0.832
	Medium	1	-	1		1	
	Maximal	1.57 (0.97;2.53)^*^	0.067	1.46 (0.94;2.27)^*^	0.090	1.61 (0.96;2.68)^*^	0.069

The GP model was adjusted for practice type, age, gender and time pressure

The staff model was adjusted for practice type, age, time pressure and occupation

^**^Statistically significant on a *p*-value level ≤ 0.05

^*^Statistically significant on a *p*-value level ≤ 0.10

### Degrees of task delegation and GPs’ job satisfaction

The analysis of associations between degrees of task delegation and GPs’ job satisfaction showed that compared to “medium degree” of task delegation, there was a tendency that “maximal degree” of task delegation was associated with overall job satisfaction (OR = 1.57, *p*-value = 0.067), satisfaction with challenges in work (OR = 1.46, *p*-value =0.090) and satisfaction with the working environment (OR = 1.61, *p*-value =0.069) (see Table 4).

## Discussion

We found a significant association between perceived “maximal degree” of task delegation in management of patients with COPD and the staff’s overall job satisfaction. The odds ratio of the staff’s satisfaction with the working environment displayed a tendency that there is also an association with “maximal degree” of task delegation. We did not find significant associations between degrees of task delegation and satisfaction of the general practitioners. However, the odds ratios of the results indicate that there is a tendency that “maximal degree” of task delegation is associated with overall job satisfaction, satisfaction with the challenges in work, and satisfaction with the working environment.

Thus, a high degree of task delegation might be one explanation for the GPs’ and their staff’s job satisfaction, which possibly confirms the part of the conceptual framework of Hackman and Oldham’s model saying that the freedom of scheduling and performing one’s own work might advance a psychological state of experienced responsibility for outcomes of the work which contributes to job satisfaction [19].

These results are also consistent with findings from previous research [13–17]. However, in these studies, the study sample was modest making it difficult to generalise the findings. Nevertheless, our results of the GP analysis displaying the tendency that they are more satisfied in the group with perceived “maximal degree” of task delegation are supported by another study showing that GPs generally would like their staff to be more involved in clinical work [23].

Associations between task delegation and healthcare professionals’ job satisfaction have been examined in other healthcare settings as well. Hence, a study conducted in two hospitals found that one of the strongest predictors of job satisfaction among staff nurses was task delegation, a finding which is further underpinning our results [24]. However, none of the mentioned studies have explicitly investigated the influence of degrees of task delegation on job satisfaction, and to our knowledge, we are the first to do that.

### Strengths and limitations of the study

The Organisation of General Practitioners in Denmark provided email addresses on 3440 GPs in Denmark corresponding to approximately 96% of the entire population of GPs. Hence, we were able to invite nearly all Danish GPs, and subsequently their staff, to participate in the survey which is a major strength of our study, and the GP survey obtained a satisfactory response rate giving a certain weight to the results.

Moreover, we used data from the Organisation of General Practitioners (PLO) and tested the group of respondents among the GPs for representation of age, gender and practice type. We found an overall resemblance to the entire study population which encompassed the 96% of all Danish GPs who were then registered with an email address at the PLO. However, a difference was found in the age group “≥65 years”, where the proportion of respondents was underrepresented (6.7% compared to 12.1%), and in practice type, with partnership practices being overrepresented (74.5% compared to 65.1%). We were not able to run similar tests of representation on the results of the staff since we did not have the corresponding information on the entire staff population. Table 5 shows descriptive statistics on GPs and staff.

**Table 5:** Distribution of healthcare professionals on practice characteristics

Practice characteristics	Number of healthcare professionals	
	GPs	Staff
	*N* (%)	*N* (%)
Practice type		
Singlehanded	198 (15.9%)	91 (14.4%)
Partnership	1047 (84.1%)	540 (85.6%)
Gender		
Male	597 (48.0%)	n.a.^a^
Female	648 (52.0%)	n.a.^a^
Age		
≤34		26 (4.1%)
35–44	291 (23.4%)	185 (29.3%)
45–54	393 (31.6%)	262 (41.5%)
55–64	477 (38.3%)	154 (24.4%)
≥65	84 (6.7%)	4 (0.6%)
Time pressure		
Very often	332 (26.7%)	29 (4.6%)
Often	512 (41.1%)	149 (23.6%)
Sometimes	346 (27.8%)	355 (56.3%)
Not so often	55 (4.4%)	98 (15.5%)
Occupation		
Nurse	n.a.	444 (70.4%)
Medical laboratory technician	n.a.	30 (4.8%)
Healthcare worker	n.a.	24 (3.8%)
Secretary	n.a.	109 (17.3%)
Other	n.a.	24 (3.8%)
Total	1245 (100)	631 (100%)

^a^Not included in the questionnaire since nurses, who are the predominant part of the staff, comprise only 3% males on a national level, and the other occupations are female dominated as well

It was also a strength that, unlike previous research, this study was conducted within everyday care reflecting perceptions of general practice as it generally is and not merely in relation to an intervention [14] or a new permanent working structure assessed within a short time frame [15].

A weakness of the study is the method used to identify members of the staff performing clinical tasks regarding COPD. The number of personal codes was sent out to the staff in each practice according to the number of staff members stated by the GPs who had participated in the GP survey. Since the GPs within the same practice did not necessarily agree on this number, we chose to send out the highest number stated to be absolutely sure that all relevant staff members would be able to respond to the questionnaire.

This method implied the risk of distributing too many personal codes to “non-existing” staff members in the concerned practices, and therefore it was not possible to calculate a reliable response rate for the staff questionnaire. This means that the response rate most likely is considerably higher than it appears (30.8%).

The staff in 44.4% of the participating practices responded to the questionnaire, which is somewhat low compared to the 57.3% in the GP questionnaire. Nevertheless, seen in isolation, 44.4% is not a poor response rate.

Common to both surveys was the risk of selection bias. The high percentage of GPs as well as staff members stating to be either “satisfied” or “very satisfied” could be an effect caused partly by this circumstance. However, high satisfaction in surveys concerning general practice in Denmark is common [25] suggesting that this potential selection bias is not that pronounced in our study.

Another concern regarding our study is the definition of degrees of task delegation and thereby the categorisation of the task delegation variable. The definition was based on interviews with members of the target group who were asked to assess each task, identified from previous interviews, and explain this assessment. There is an extent of uncertainty attached to our categorisation, and it is therefore open for discussion. However, we asked six persons to carry out this assessment, three of them being GPs delegating the tasks, and three of them being the ones conducting them. Their overall agreement on the definition of the tasks enhances our results.

Also the decision to use three tasks as the threshold for categorising the respondents into either category 1 or category 2 should be addressed as a concern regarding development of the task delegation variable. Since we were not able to find an existing categorisation for our purpose, this threshold is arbitrary and open for discussion as well. However, we have tried to change the threshold from three to four tasks, and this change enhanced our results making them significant on a 5% *p*-value level. This sensitivity analysis is displayed in the Additional file 3.

A confounding factor regarding the GP analysis is the fact that Danish GPs are self-employed meaning that they have the authority to organise their practice in the way that suits them the best. However, they are working within the frames of a contract with the public funder, which may put a limit to the freedom of organising, forcing them to make undesirable decisions. Hence, endogeneity may be minimised by these contextual circumstances.

Even though the analyses displayed that there is a significant association between “maximal degree” of task delegation and overall job satisfaction of the staff and a tendency of this association regarding the GPs, the ORs differed considerably between the two analyses. Hence, in the staff analysis the OR was 1.88, while in the GP analysis, the OR was 1.57. The reason for this difference in the results could be that the GPs might perceive it as a loss of control when delegating tasks to their staff. Thus, it has been suggested that in order to maintain their own position as the medical experts, GPs try to control nurses’ activities despite the fact that they acknowledge their importance to the provision of healthcare [26, 27].

### Implications

Medical as well as perceived quality of care is essential to the patients, and since job satisfaction is associated with these outcomes [9, 11], it should be taken into consideration when (re) organising general practice. The finding that there is a significant association between a high degree of task delegation and the staff’s overall job satisfaction show that GPs should not be reluctant to delegate tasks considering the influence on their job satisfaction. It is possible, that there might be positive associations between a high degree of task delegation and job satisfaction of GPs as well, even though we have only found tendencies in this analysis. Importantly, the results indicate that no negative associations between these variables exist.

This knowledge can be used to inform GPs and practice managers in future delegation processes within general practice and may also be valuable for policy makers in the decision making regarding the overall organisation of healthcare in the future. However, further research is needed to confirm the tendencies found in our study.

## Conclusions

We conclude that a high degree of task delegation is significantly associated with overall job satisfaction of the staff, and that there is a tendency that a high degree of task delegation is associated with the GPs’ and the staff’s satisfaction with the working environment as well as with GPs’ overall job satisfaction and satisfaction with challenges in work. To qualify future delegation processes within general practice, further research could explore the reasons for our findings.

## Additional files

Additional file 1: The task delegation questions as they were presented in the questionnaires. (DOCX 36 kb)
Additional file 2: Tasks identified through interviews. (DOCX 15 kb)
Additional file 3: Sensitivity of the results using four tasks rated as 2 instead of three for categorising respondents into “medium degree” of delegation. (DOCX 20 kb)

## Abbreviations

CI: Confidence interval
COPD: Chronic obstructive pulmonary disease
GP: General practitioner
OR: Odds ratio
