# Supplementary material for: Associations between degrees of task delegation and job satisfaction of general practitioners and their staff: a cross-sectional study
Source: BMC Health Serv Res. 2017 Jan 17;17:44. doi: 10.1186/s12913-017-1984-y (PMC5240386; doi:10.1186/s12913-017-1984-y)
Supplement: Additional file 1: — The task delegation questions as they were presented in the questionnaires. (DOCX 36 kb) [file 12913_2017_1984_MOESM1_ESM.docx]

**We will now ask you some questions, which concern task delegation regarding management of patients with COPD in your practice**

The questions are about, who undertakes various tasks in each of the following types of consultations with COPD patients:

- Diagnosis
- Annual follow-up
- Semiannual and quarterly follow-up
- Exacerbations

Who is **typically** undertaking the following task regarding **diagnosis** of patients with COPD in your practice?

*Select one or more answers*

|  | GP, including GP trainee | Nurse | Medical laboratory technician | Secretary or other staff member | Not performed/  Performed elsewhere | Do not know |
| --- | --- | --- | --- | --- | --- | --- |
| Performing spirometry |  |  |  |  |  |  |
| Recording patient’s medical history |  |  |  |  |  |  |
| Drawing blood samples |  |  |  |  |  |  |
| Measuring oxygen saturation |  |  |  |  |  |  |
| Instructing in inhalation technique |  |  |  |  |  |  |
| Measuring inspiratory flow |  |  |  |  |  |  |
| Assessment of needs for initiating or adjusting COPD medication |  |  |  |  |  |  |
| Assessment of functional level – e.g. MRC scale |  |  |  |  |  |  |
| Assessment of quality of life, e.g. CAT-score |  |  |  |  |  |  |
| Assessment of sputum, e.g. according to the patient’s description |  |  |  |  |  |  |
| Counseling with regard to self-care |  |  |  |  |  |  |
| Measuring blood pressure |  |  |  |  |  |  |

Who is **typically** undertaking the following task regarding **annual follow-up** of patients with COPD in your practice?

*Select one or more answers*

|  | | GP, including GP trainee | | Nurse | Medical laboratory technician | Secretary or other staff member | | Not performed/  Performed elsewhere | | Do not know | |
| --- | --- | --- | --- | --- | --- | --- | --- | --- | --- | --- | --- |
| Performing spirometry |  | |  | |  | |  | |  | |  |
| Reviewing history of disease progression, e.g. number of exacerbations within the last year |  | |  | |  | |  | |  | |  |
| Drawing blood samples |  | |  | |  | |  | |  | |  |
| Measuring oxygen saturation |  | |  | |  | |  | |  | |  |
| Instructing in inhalation technique |  | |  | |  | |  | |  | |  |
| Measuring inspiratory flow |  | |  | |  | |  | |  | |  |
| Reviewing patients’ COPD medication |  | |  | |  | |  | |  | |  |
| Assessment of functional level, e.g. MRC scale |  | |  | |  | |  | |  | |  |
| Assessment of quality of life, e.g. CAT-score |  | |  | |  | |  | |  | |  |
| Assessment of sputum, e.g. according to the patient’s description |  | |  | |  | |  | |  | |  |
| Counseling with regard to self-care |  | |  | |  | |  | |  | |  |
| Counseling with regard to vaccination against influenza and pneumonia |  | |  | |  | |  | |  | |  |
| Counseling with regard to smoking cessation |  | |  | |  | |  | |  | |  |
| Counseling with regard to diet and exercise |  | |  | |  | |  | |  | |  |
| Measuring blood pressure |  | |  | |  | |  | |  | |  |
| Performing echocardiography | |  |  | |  | |  | |  | |  |

Who is **typically** undertaking the following task regarding **semiannual and quarterly follow-up** of patients with COPD in your practice?

*Select one or more answers*

|  | | GP, including GP trainee | | Nurse | Medical laboratory technician | Secretary or other staff member | | Not performed/  Performed elsewhere | | Do not know | |
| --- | --- | --- | --- | --- | --- | --- | --- | --- | --- | --- | --- |
| Performing spirometry |  | |  | |  | |  | |  | |  |
| Drawing blood samples |  | |  | |  | |  | |  | |  |
| Measuring oxygen saturation |  | |  | |  | |  | |  | |  |
| Instructing in inhalation technique |  | |  | |  | |  | |  | |  |
| Measuring inspiratory flow |  | |  | |  | |  | |  | |  |
| Assessment of needs for initiating or adjusting COPD medication |  | |  | |  | |  | |  | |  |
| Assessment of functional level, e.g. MRC scale |  | |  | |  | |  | |  | |  |
| Assessment of quality of life, e.g. CAT-score |  | |  | |  | |  | |  | |  |
| Assessment of sputum, e.g. according to the patient’s description |  | |  | |  | |  | |  | |  |
| Counseling with regard to self-care |  | |  | |  | |  | |  | |  |
| Counseling with regard to vaccination against influenza and pneumonia |  | |  | |  | |  | |  | |  |
| Counseling with regard to smoking cessation |  | |  | |  | |  | |  | |  |
| Counseling with regard to diet and exercise |  | |  | |  | |  | |  | |  |
| Measuring blood pressure |  | |  | |  | |  | |  | |  |

Who is **typically** undertaking the following task regarding **exacerbations** in patients with COPD in your practice?

*Select one or more answers*

|  | GP, including GP trainee | Nurse | Medical laboratory technician | | Secretary or other staff member | | Not performed/  Performed elsewhere | | Do not know | |  |
| --- | --- | --- | --- | --- | --- | --- | --- | --- | --- | --- | --- |
| Performing stethoscopy |  |  | |  | |  | |  | |  | |
| Drawing blood samples, e.g. CRP |  |  | |  | |  | |  | |  | |
| Measuring oxygen saturation |  |  | |  | |  | |  | |  | |
| Assessment of needs for initiating or adjusting COPD medication |  |  | |  | |  | |  | |  | |
| Assessment of sputum, e.g. according to the patient’s description |  |  | |  | |  | |  | |  | |
| Assessment of indication for use of antibiotics |  |  | |  | |  | |  | |  | |
| Assessment of indication for use of prednisolone |  |  | |  | |  | |  | |  | |
| Counseling with regard to self-care |  |  | |  | |  | |  | |  | |
