# Supplementary material for: Associations between degrees of task delegation and job satisfaction of general practitioners and their staff: a cross-sectional study
Source: BMC Health Serv Res. 2017 Jan 17;17:44. doi: 10.1186/s12913-017-1984-y (PMC5240386; doi:10.1186/s12913-017-1984-y)
Supplement: Additional file 2: — Tasks identified through interviews. (DOCX 15 kb) [file 12913_2017_1984_MOESM2_ESM.docx]

Tasks identified through interviews

| **Clinical task** |
| --- |
|  |
| Drawing blood samples |
| Measuring blood pressure |
| Measuring oxygen saturation |
| Performing spirometry |
| Instructing in inhalation technique |
| Measuring inspiratory flow |
| Recording patient’s medical history |
| Performing echocardiography |
| Counselling with regard to self-care |
| Counselling with regard to vaccination against influenza and pneumonia |
| Counselling with regard to smoking cessation |
| Counselling with regard to diet and exercise |
| Assessment of sputum, e.g. according to the patient’s description |
| Assessment of functional level – e.g. using an MRC scale |
| Assessment of life quality, e.g. CAT-score |
| Assessment of needs for initiating or adjusting COPD medication |
| Assessment of indication for use of prednisolone |
| Assessment of indication for use of antibiotics |
| Performing stethoscopy |
