# Supplementary material for: Associations between degrees of task delegation and job satisfaction of general practitioners and their staff: a cross-sectional study
Source: BMC Health Serv Res. 2017 Jan 17;17:44. doi: 10.1186/s12913-017-1984-y (PMC5240386; doi:10.1186/s12913-017-1984-y)
Supplement: Additional file 3: — Sensitivity of the results using four tasks rated as 2 instead of three for categorising respondents into “medium degree” of delegation. (DOCX 20 kb) [file 12913_2017_1984_MOESM3_ESM.docx]

**Sensitivity of results using four tasks rated as 2 instead of three for categorising respondents into “medium degree” of delegation**

| **Degree of delegation**  **Staff** | **Job satisfaction** | | | | | |  |
| --- | --- | --- | --- | --- | --- | --- | --- |
|  | **Overall job satisfaction** | | **Challenges in work** | | **Working environment** | |  |
|  | **OR adj. (95% CI)** | **p** | **OR adj. (95% CI)** | **p** | **OR adj. (95% CI)** | **p** |  |
| **3 tasks** | | | | | | |  |
| Minimal | 1.23 (0.75;2.02) | 0.401 | 0.98 (0.62;1.55) | 0.935 | 2.21 0.62;7.86 | 0.222 |  |
| Medium | 1 | - | 1 | - | 1 | - |  |
| Maximal | 1.88 (1.00;3.51)* | 0.048 | 1.26 (0.71;2.21) | 0.431 | 4.33 (0.78;24.08)** | 0.094 |  |
| **4 tasks** | | | | | | |  |
| Minimal | 1.45 (0.83;2.51) | 0.189 | 1.12 (0.67;1.87) | 0.652 | 2.33 (0.60;9.05) | 0.222 |  |
| Medium | 1 | - | 1 | - | 1 | - |  |
| Maximal | 2.17 (1.09;4.31)* | 0.027 | 1.39 (0.75;2.58) | 0.300 | 4.85 (0.80;29.59)** | 0.087 |  |
|  | | | | | | |  |
| **Degree of delegation**  **GPs** | **Job satisfaction** | | | | | |  |
|  | **Overall job satisfaction** | | **Challenges in work** | | **Working environment** | | |
|  | **OR adj. (95% CI)** | **p** | **OR adj. (95% CI)** | **p** | **OR adj. (95% CI)** | **p** | |
| **3 tasks** | | | | | | |  |
| Minimal | 1.08 (0.80;1.45) | 0.618 | 1.00 (0.76;1.31) | 0.976 | 1.03 (0.75;1.42) | 0.832 | |
| Medium | 1 | - | 1 | - | 1 | - | |
| Maximal | 1.57 (0.97;2.53)** | 0.067 | 1.46 (0.94;2.27)** | 0.090 | 1.61 (0.96;2.68)** | 0.069 | |
| **4 tasks** | | | | | | |  |
| Minimal | 1.15 (0.82;1.61) | 0.423 | 1.23 (0.90;1.68) | 0.189 | 1.09 (0.77;1.56) | 0.620 | |
| Medium | 1 | - | 1 | - | 1 | - | |
| Maximal | 1.66 (1.00;2.75)** | 0.051 | 1.74 (1.09;2.78)* | 0.019 | 1.69 (0.98;2.90)** | 0.058 | |

* Statistically significant on a p-value level ≤ 0.05
** Statistically significant on a p-value level ≤ 0.10

|  | |  |  |
| --- | --- | --- | --- |
|  |  | |  |
